# Vitamin K and Bone Health: A Review on the Effects of Vitamin K Deficiency and Supplementation and the Effect of Non-Vitamin K Antagonist Oral Anticoagulants on Different Bone Parameters

**DOI:** 10.1155/2019/2069176

**Published:** 2019-12-31

**Authors:** Celia Rodríguez-Olleros Rodríguez, Manuel Díaz Curiel

**Affiliations:** Internal Medicine, Bone Disease Department, Hospital Universitario Fundación Jiménez Díaz, Av. de los Reyes Católicos, 2, 28040 Madrid, Spain

## Abstract

Although known for its importance in the coagulation cascade, vitamin K has other functions. It is an essential vitamin for bone health, taking part in the carboxylation of many bone-related proteins, regulating genetic transcription of osteoblastic markers, and regulating bone reabsorption. Vitamin K deficiency is not uncommon, as deposits are scarce and dependent upon dietary supplementation and absorption. Vitamin K antagonist oral anticoagulants, which are prescribed to many patients, also induce vitamin K deficiency. Most studies find that low serum K1 concentrations, high levels of undercarboxylated osteocalcin (ucOC), and low dietary intake of both K1 and K2 are associated with a higher risk of fracture and lower BMD. Studies exploring the relationship between vitamin K supplementation and fracture risk also find that the risk of fracture is reduced with supplements, but high quality studies designed to evaluate fracture as its primary endpoint are needed. The reduction in risk of fracture with the use of non-vitamin K antagonist oral anticoagulants instead of warfarin is also of interest although once again, the available evidence offers disparate results. The scarce and limited evidence, including low quality studies reaching disparate conclusions, makes it impossible to extract solid conclusions on this topic, especially concerning the use of vitamin K supplements.

## 1. Aim of the Review

The following narrative review aims at summarizing the most relevant and current evidence concerning the relationship between vitamin K and bone, exploring the links between both, and the effect of the deficiency and supplementation of vitamin K on different bone parameters. Special attention was given to the bone-safety profile of non-vitamin K antagonist oral anticoagulants (NOACs). We aimed at investigating whether the available evidence is solid and reliable enough for extracting practice-changing recommendations.

## 2. Materials and Methods

The search terms “Vitamin K” and “Bone” were introduced in PubMed, Medline, and Cochrane databases. Also, the names of the different NOACs and the term “Bone” were introduced. Only papers written in English were reviewed. Articles exploring relevant aspects of the relationship between vitamin K and bone were included, specifically, articles investigating the biochemical link between both and the effect of vitamin K deficiency (including the use of vitamin K antagonists) and supplementation on bone health, expressed as different biochemical markers, analytical values such as bone mineral density (BMD), and clinical outcomes (fractures).

## 3. Vitamin K: General Concepts

### 3.1. Types of Vitamin K

Vitamin K is a family of fat-soluble compounds that share a 2-methyl-1,4-naphthoquinone structure called menadione and a variable side chain at the 3-position [1, 2]. The latter defines the three main types of vitamin K: vitamin K1 or phylloquinone (PK), vitamin K2 or menaquinones (MKs), and vitamin K3 or menadione.

PK, characterized by a phytyl side chain, is the major dietary source of vitamin K in Western diets [3]. It is the form of vitamin K synthesized by plants and cyanobacteria, and it is mainly found in green leafy vegetables although other foods, such as other vegetables (including vegetable-derived oils), fruits, grains, and dairy, carry a significant amount of this compound [1].

Vitamin K2 or MK is synthesized by certain bacteria, which produce a polymer of repeating prenyl units as a side chain. In fact, MKs are classified according to the number of prenyl units into 13 subtypes (MK-2 to MK-14). Most of these prenyl units are unsaturated, but some bacteria produce saturated units, thus adding extra hydrogen atoms to the MK subtype (MK-n (Hn)). With the exception of MK-4, all MK subtypes are synthesized by bacteria present in the human gut (mainly bacteroides in the large intestine, which mostly produce MK 10–13) [4] or bacteria present in some foods including animal liver and fermented foods (mainly cheese in Western countries, and natto, a soybean-based food in Japan). Natto is especially rich in MK-7, which has the highest bioactivity and half-life compared to PK and MK-4 [5, 6]. Long-chain MK produced by enteric flora have low bioactivity.

On the contrary, MK-4 is produced from PK in a two-phase process. Firstly, PK is converted to menadione in specific tissues (testes, pancreas, and vessel wall). Menadione is a water-soluble type of vitamin K lacking a side chain. It is also a synthetic analog added to animal food. Menadione is converted to MK-4 in the liver. It has also been suggested that vitamin K2 subtypes with longer chains can be converted to MK-4 [4]. MK-4 is the predominant form of vitamin K in the human body.

### 3.2. Metabolism

In the presence of normal biliopancreatic function, vitamin K1 is absorbed in the small bowel, while vitamin K2 is absorbed in the colon [2]. Both are transported in triglyceride-rich chylomicrons in the lymphatic system [7]. Most of vitamin K1 stays in the liver, but a small part flows back into the circulation and is carried by very-low-density lipoproteins (VLDL) to extrahepatic tissues [7]. MKs are carried by low-density lipoproteins (LDL) to extrahepatic tissues. The exception is MK-4, which can be transported by either high- or low-density lipoproteins [7].

Only small amounts of vitamin K are stored in the body [8]. Tissue distribution varies [2]; main stores of PK are found in the liver, heart, and pancreas, whereas long-chain MKs are found in the liver and MK-4 in the pancreas, brain, and lung [4].

### 3.3. Deficiency and Supplements

Up to date, there is not enough evidence to determine the estimated average requirement for vitamin K [5], and consequently, recommendations are inconsistent. This difficulty is partly due to significant variation in the correlation between vitamin K levels and concentrations of carboxylated vitamin K-dependent proteins, a difficulty derived from genetic polymorphisms in enzymes such as vitamin K-dependent gamma-glutamyl carboxylase (GGCX) and for vitamin K epoxide reductase complex subunit 1 gene (VKORC1) [5].

For instance, the Health and Medicine Division set in 2001 Adequate Intake (AI) values based on median intake values reported by the National Health and Nutrition Examination Survey (NHANES) III; the AI for vitamin K1 for adult females was set at 90 *μ*g/dl and 120 *μ*g/dl for adult males [9]. There is no solid evidence supporting that these amounts are enough for maintaining carboxylation of vitamin K-dependent proteins. Hepatic vitamin K stores are destined for maintaining carboxylation of coagulation-related proteins in the event of short-term dietary depletion (one month approximately), but not carboxylation of other proteins, which may require larger doses for their carboxylation to be preserved [5]. To this moment, the Health and Medicine Division has not established the value of AI for MK even though certain subtypes of MK have a higher bioavailability than PK.

Vitamin K deficiency may occur as a result not only of an inadequate dietary supply but also because of many health problems, including liver disease, biliopancreatic disturbances, cystic fibrosis, alcoholism, or enteric diseases that may cause malabsorption (inflammatory bowel disease, short bowel syndrome, etc.) [10]. Most importantly, some medications are also a cause of vitamin K depletion. In this sense, the use of vitamin K antagonist oral anticoagulants (VKAs) deserves special attention because of current evidence suggesting NOACs to have a more bone-friendly profile.

Vitamin K1, MK-4, and MK-7 oral supplements are available [11]. K1 supplements are the most frequently used, not only for preventing deficiency but also to solve coagulation problems caused by anticoagulant poisoning or other diseases. Food supplements of Mk-4 and Mk-7 (natto derived) are available. MK-4 has a similar molecular structure than K1, but MK-7 has a longer side chain and thus has the highest bioavailability and steady blood levels throughout time [4]. A paper comparing pharmacodynamic and pharmacokinetic differences between K1 and Mk-7 supplements interestingly found that Mk-7 induced more complete carboxylation of osteocalcin, suggesting higher effectiveness [11]. The explanation for this finding does not only rely on its longer half-life but also, as authors suggest, to a higher activity of Mk-7 as a cofactor found in another study [12]. In Japan, vitamin K2 supplements are an approved treatment for osteoporosis [13].

Adverse reactions are rare, but there have been some reports of minor skin and gastrointestinal reactions [14, 15]. Some studies have also reported potential benefit of K1 intake and lipid profile, insulin sensitivity, and glucose levels [16, 17]. Menadione supplements have not been approved to date due to pending safety issues.

## 4. Vitamin K and Bone

### 4.1. Mechanisms of Action

Vitamin K acts in the bone by several mechanisms. Firstly and most well-known, vitamin K is an essential coenzyme for the gamma-glutamyl carboxylase enzyme, which carboxylates glutamic acid (Glu) residues in vitamin K-dependent proteins, transforming them into gamma-carboxyglutamic acid (GIa). There are several vitamin K-dependent proteins in the bone, including matrix G1a protein, periostin, gas 6, protein S, and osteocalcin (or bone Gla protein) [4]. Osteocalcin is synthesized by osteoblasts during the mineralization phase of the bone, and it binds to calcium ions and hydroxyapatite crystals, regulating their size and shape [4]. It has three Glu residues, and its binding capacity depends on its degree of carboxylation. MK supplements effectively rise carboxylated osteocalcin concentrations. However, full carboxylation of Glu residues is not the normal state of osteocalcin in human bone tissue and must not be the aim with supplementation [4, 5]. Also, in the past years, osteocalcin has been discovered to play an unexpected role in the regulation of energy metabolism, and bone has been marked as an important endocrine organ [18]. After binding to its receptor in osteoblasts, insulin promotes decarboxylated osteocalcin formation [19]. The latter appears to be the molecule behind these endocrine functions, as it has been proven to alter pancreatic beta cell production, and insulin secretion, sensitivity, and expression [18]. Indeed, mice lacking insulin receptor in osteoblasts suffer from glucose intolerance [18], thus demonstrating the importance of the role of bones in glucose homeostasis. On the contrary, insulin itself plays an important role in bone metabolism and is needed for normal postnatal bone acquisition [20] and the regulation of bone resorption through its action on both osteoclasts and osteoblasts. A connection between both processes, i.e., insulin-bone health and insulin-descarboxylated osteocalcin, was later found; bone resorption enhanced by insulin generates a low pH which is sufficient to promote extracellular osteocalcin decarboxylation, which in turn exerts endocrine function [19].

Another vitamin K-dependent protein is matrix G1a protein, which is secreted by chondrocytes and vascular smooth cells, and exerts its role as an inhibitor of angiogenesis and ectopic tissue calcification [21]. MGP knockout mice show dysregulation of endothelial differentiation conducing vascular abnormalities such as arteriovenous malformations and arterial calcification (with increased rupture risk) [21]. In fact, high levels of undercarboxylated matrix G1a protein are considered a marker of cardiovascular disease [22]. G1a-rich protein and periostin regulate extracellular matrix mineralization, and protein S, although mainly known for its role in coagulation, also plays a role in bone turnover although its pathways are unclear.

In addition to gamma-carboxylation, vitamin K plays an important role in bone via other mechanisms. It can regulate genetic transcription of osteoblastic markers, can suppress bone resorption, and can regulate the formation of osteoclasts [2]. In vitro and animal studies have shown that MK-4 may be involved in inflammation [23], oxidative stress, and apoptosis, all of which can inhibit bone reabsorption. An in vitro study showed that MK-7 suppressed osteoblast differentiation and stimulated mRNA production of osteocalcin, osteoprotegerin, and RANK-L [24]. Vitamin K2 also activates the orphan nuclear steroid and xenobiotic receptor (SXR), inducing expression of its target genes: CYP-450 (mainly CYP3A4 and CYP2C8) and ATP transport proteins (such as MDR1 and MRP2) [25]. SXR is also involved in expression of osteoblastic markers, favoring bone formation [4].

### 4.2. Vitamin K, Bone Mineral Density (BMD), and Fractures

Most studies find that low serum K1 concentrations, high levels of undercarboxylated osteocalcin (ucOC), and low dietary intake of both K1 and K2 are associated with a higher risk of fracture.

In relation to dietary intake of vitamin K and fracture risk, evidence is substantial. One of the largest studies in this matter is a prospective analysis conducted within the *Nurse' Health Study* [26], performed in 72.327 women between 38 and 74 years of age, with a 10-year follow-up. In this study, subjects with a vitamin K1 intake more than 109 *μ*g/day presented a significant lower age-adjusted relative risk of hip fracture than women with a lower intake (RR: 0.70; 95% confidence interval (CI): 0.53, 0.93). No benefit in fractures was found with higher intakes; it rather appeared to exist a threshold upon which fracture risk began to rise. A more recent meta-analysis [27] including 4 cohort studies and 1 nested case-control study, summing a total of 80,982 participants and 1114 fractures showed an inverse association between dietary vitamin K1 intake and risk of fractures, finding no “threshold effect.” In this study, subjects with highest intake of vitamin K presented a 22% reduction in fracture risk (95% CI: 0.56–0.99), but it is worth mentioning that moderate heterogeneity was found between studies. Subgroup analysis showed that only studies with a follow-up of 10 or more years found this association.

Whether higher vitamin K intakes are associated with higher BMD values is still a controversial matter. Although dietary intake has been linked to fracture risk, results on the effect on BMD are more inconsistent [28]. For instance, using the *Framingham study cohort*, Booth et al. [29] investigated the change in BMD in 6 anatomic sites in 888 patients with a mean age of 75 years with different PK intakes evaluated by a validated food frequency questionnaire. The authors did not find a significant association between BMD at any site and PK intake despite correcting for potential confounding variables such as age, body mass index, smoking/alcohol use, other dietary intakes (calcium and vitamin D), and estrogen use. However, they did find significant association between PK intake and incident fractures, suggesting this event was mediated by factors other than BMD loss. The same authors published three years after an analysis of the Framingham cohort [30], this time including 2591 individuals, with a younger mean age (58 and 59 years for women and men, respectively). In this analysis, low PK intake did show an age-independent association with BMD at the hip and spine, but only in women. The authors cannot justify these findings with the age difference between the two cohorts, but rather suggest that the higher number of participants may account for the change in results. Furthermore, a study following a cohort of 2016 Danish perimenopausal women was conducted by Rejnmark et al. [31] found no association between K1 intake and BMD at the femoral neck or lumbar spine. This study highlighted the difference in vitamin K intake in comparison to other studies (it was one-third of the intake reported by the Nurse's Health Study conducted in the United States) and the difficulty to assess this intake by food questionnaires. It suggests that the optimal way of quantifying this intake may have to involve direct testing of the food, but then again, no study makes this kind of measurements.

Overall, it is clear that occult reasons may be behind the contradictory results in the evidence concerning the effects of vitamin K intake and other bone parameters. The influence of changing dietary patterns through life and its effect on BMD is one of them. More importantly, the limitations of the tools employed to assess vitamin K intake is an important matter; although many studies use validated food questionnaires, these cannot replace objective measurements of PK in food. These studies also limit themselves in assessing K1 intake because it is the main form present in our diet, but vitamin K2 intake may also be an important variable.

Another difficulty is vitamin K intakes may correlate poorly with changes in serum concentrations and have a different magnitude on effect. This is illustrated by the following studies that explore the relationship between levels of serum vitamin K and ucOC values and BMD. Booth et al. [32] used again the *Framingham Heart study* cohort to assess vitamin K status through plasma K1 values and serum ucOC values. In this study involving 863 women and 741 men with no significant differences in mean K1 intake (151–177 *μ*g/day), poor vitamin K status was associated with low BMD at the femoral neck in men and low BMD in the spine in women without estrogen replacement therapy. This association was not significant in premenopausal or postmenopausal women with estrogen replacement therapy. Thus, this study highlights the importance of adjusting results for variables such as estrogen status. Finally, in a small study performed by Jaghsi et al. [33] in postmenopausal women without estrogen replacement therapy, serum K1 was positively correlated with lumbar spine BMD. Diagnostic sensitivity and specificity of vitamin K1 values for osteoporosis was 90% and 98%, respectively, and the authors propose that serum vitamin K1 might be of value as a diagnostic tool in osteoporosis. An important limitation in these studies is the difficulty in adjusting results for confounding variables. Other nutritional components are especially difficult to adjust for; vitamin K-rich foods may also carry other bone-friendly nutrients (calcium, magnesium, etc.) which may interfere with the results. In light of the aforementioned studies, it seems that ucOC values are a good marker of bone health.

### 4.3. Effect of Vitamin K Supplementation: Fractures and BMD

The following studies are summarized in Supplementary [Supplementary-material supplementary-material-1].

Effects of vitamin K supplementation on BMD are summarized in the meta-analysis performed by Fang et al. [34], which included both healthy subjects and patients affected by primary/secondary osteoporosis. In total, 17 studies were included, 10 of which included vitamin K2 supplements (8 with MK-4 at a dose of 15–45 mg/day and 2 using MK-7 at a dose of 0.2–3.6 mg/day) and 7 studies with vitamin K1 supplementation (0.2–10 mg/day). In the general analysis including all the selected studies, the authors found that vitamin K supplementation did not significantly affect BMD (measured by weighted mean difference) at the femoral neck but did significantly increase lumbar spine BMD by 1.27% (CI 95%: 0.47–2.06) after 6–36 months of treatment. However, when subgroup analyses were performed according to the type of vitamin K administered, the effects were not significant for K1 and still remained significant for K2 (1.8% mean increase in lumbar spine BMD, CI 95%: 0.87–2.75). Other subgroup analysis revealed only significant changes in Asian population (but then again, 5 other Japanese studies included were of low quality and accounted for important heterogeneity in the Asian studies) and non-postmenopausal women. The authors are cautious of these results; many of the included studies were of low quality, and significant heterogeneity was found between these studies (pooled analysis using only high quality studies revealed nonsignificant results). Another meta-analysis [35] specifically explored the role of vitamin K2 supplements both in BMD and fracture. Including 19 studies (11 of which were not included in the meta-analysis mentioned above) with 6759 participants, the authors found that K2 supplements only improved significantly middle- and long-term vertebral BMD and long-term forearm BMD in postmenopausal women with osteoporosis. Finally, a study involving 115 Japanese postmenopausal women who were randomized to take either a supplement containing calcium and vitamin D only, with K1 or K2 compared with a group with no supplements, showed a significant increase in total BMD in all three groups compared with controls, with additional benefits for lumbar BMD in the groups who took either K1 or K2 [36]. Globally, most of the studies report positive correlation between vitamin K supplements, at least in certain subgroups. However, the results are not consistent between studies, partly due to, once again, limitations affecting the studies, namely, other covariates that have not been adjusted (basal intake of VK and other nutrients, doses of supplements, geographical differences, duration of follow-up, and quality of the studies).

Moreover, although some studies have failed to demonstrate significant changes in BMD, they have succeeded in obtaining significant results in other bone parameters. This is the case of the study by Knapen et al. [37], in which 325 nonosteoporotic postmenopausal women receive either 45 mg/day MK-4 or placebo for three years. The authors suggest that other bone parameters must be analyzed, for BMD only does not take into account the geometry (size and thickness) of the bone, which independently affects bone strength and fracture risk. The results in this study, for instance, showed that although MK-4 supplementation did not significantly increase BMD at the hip, bone mineral content and femoral neck width were both significantly increased. Hip bone strength remained stable in the treatment group but significantly decreased in the placebo group. Another study was conducted supporting this idea by the same authors. Finally, a different study by Knapen et al. [38] in 244 healthy postmenopausal women treated with Mk-7 supplements showed a significant decrease in BMD decline at the lumbar spine and femoral neck, but not at total hip, and increased bone strength (measured as compression, bending, and impact strength).

With regard to fractures, another systematic review analyzed both BMD and fracture risk with K1 or MK-4 supplements [15]. The authors found that all thirteen trials except one showed a decrease in bone loss (measured by BMD) in patients supplemented with either type of vitamin K. Most importantly, they analyzed fracture data in 7 trials (all but 2 using MK-4 and mostly conducted in Japanese postmenopausal women) and found that MK-4 supplements caused a reduction in all fracture types absolute difference in fracture rates: hip 6% (95% CI 3–9%), vertebral 13% (95% CI 6–21%), and all nonvertebral fractures 9% (95% CI 6–12%), with no significant heterogeneity between studies. Limitations are that most of these studies were not specifically designed to assess fracture as the primary endpoint, that some of the studies were not of high quality, and that although homogeneity for certain variables was assured, studies might vary in other factors such as population characteristics and other cosupplements used.

In contrast, a study comparing the effect of three-year calcium monotherapy or bitherapy with MK-4 in 4378 Japanese osteoporotic postmenopausal women found no benefit in the incidence rate of new vertebral fractures in the bitherapy group [39]. Only post hoc analysis showed a reduction in vertebral fractures in women who had 5 or more prevalent fractures. However, in this study, a possible confounding factor is that other treatments aimed at treating osteoporosis could be administered without restriction; treatment variability could interfere in results.

Finally, a specific study addressing K1 supplementation and fracture risk is mentioned. Cheung et al. [13] performed a randomized controlled trial (RCT) in 440 postmenopausal Canadian women with osteopenia. The results revealed that K1 5 mg/day supplements reduced clinical vertebral fractures (9 versus 20, p 0.04) although this was not the primary outcome of the study. This study failed to demonstrate a change in BMD at the lumbar spine or total hip and, as mentioned before, suggests that vitamin K effects on bone are not entirely BMD-related.

All studies considered, we can conclude that supplementation with vitamin K seems to reduce fractures, but that a large, high quality, and fracture-based study is needed to confirm these results in order to make a specific, practice-changing recommendation.

### 4.4. Effect of Vitamin K Supplements in Association with Other Treatments Aimed at Treating Osteoporosis

Evidence on this matter is scarce, especially concerning the effect on fractures. The largest study addressing this issue is a RCT involving 1874 women aged 65 or more with osteoporosis, who received either bitherapy with risedronate and vitamin K2 or risedronate alone [14]. Incidence rates of fracture were similar between the two groups, and subgroup analysis failed to demonstrate differences when patients were stratified upon ucOC serum values.

A smaller study [40] in 101 women comparing risedronate and K2 bitherapy versus monotherapy with risedronate failed again to demonstrate a reduction in vertebral fracture incidence between the two groups but found that ucOC levels were greater in patients with vertebral fractures treated with risedronate alone, in comparison with bitherapy. Finally, a small study [41] involving 62 postmenopausal women with rheumatoid arthritis and osteopenia or osteoporosis found that combined therapy with alendronate and vitamin K2, decreases ucOC and bone metabolism markers and increases BMD in both lumbar spine and femoral neck.

In light of these contradicting results, vitamin K supplements cannot be recommended for osteoporosis treatment, at least in individuals who are not at risk of vitamin K deficiency for specific reasons.

## 5. NOACs and Fractures

VKA causes vitamin K deficiency by blocking the enzyme vitamin K epoxide reductase, thus depleting vitamin K hydroquinone which is essential for the activity of glutamyl carboxylase and hence posing a potential threat to bone health through this mechanism [42]. However, the available evidence on the effect of VKA on fracture offers disparate results, with some studies reporting increased fracture risk at different sites (except hip) [43–45] and others not [46–48]. These findings may be a consequence of the limitations of these studies [49], for instance, a short follow-up of the individuals included in them or the evaluation of fractures only in certain sites.

NOAC prescription has dramatically increased in the past few years as an alternative to VKAs [49], and in contrast to the latter, NOACs do not interfere with the vitamin K cycle. Evidence exploring their bone-safety profile has been published, both in rats [50–53] and humans [49, 54–58].

Studies exploring effect of NOACs in rats suggest a favorable bone-safety profile; rivaroxaban proved not to interfere with healing of rat femur fractures [50], and edoxaban did not interfere with total G1a-osteocalcin levels [51]. In a study by Prodinger et al. [53], both rivaroxaban and enoxaparin induced morphological changes in the fracture callus of 70 rats, but these alterations did not result in functional deficits. In the paper by Fusaro et al. [52] comparing a 6-week regimen of dabigatran, placebo, or warfarin, the administration of warfarin proved to reduce bone trabecular size and structure, increase bone turnover, and reduce mineralization compared to dabigatran.

Evidence concerning bone-safety profile of NOACs in humans has also been published, but there is not a single RCT evaluating this as its primary outcome.

Gu et al. [49] carried out a meta-analysis [49] that included 12 randomized control trials (RCT) comparing the efficacy of NOACS versus warfarin as their primary outcome, but that reported data on fracture too. Most of these studies were the pivotal studies of each NOAC, and none had fracture as their primary outcome and where therefore not designed for this purpose. However limited, this paper does reach interesting results: NOACs significantly reduced the risk of any fracture by 18% (RR: 0.82, 95% CI: 0.73–0.93, *P*=0.001) compared to warfarin, with a high NNT of 333. When analyzed, particular fracture sites and a composite of fragility fractures (including vertebral, hip, rib, and wrist fracture) did not yield statistically significant differences in both groups, and the authors propose a low incidence of fractures as a possible explanation. Limitations include that none of the RCTs were designed to assess fractures as its primary outcome, and that methods of data collection regarding fractures differed between studies. Also, presuming warfarin fracture-related risk is cumulative, the authors conclude that studies with short-term warfarin treatment might underestimate fracture risk. There are two additional observational studies which assess fracture risk amongst many other safety and also efficacy values. The first one is a paper by Steffel et al. [58], who carried out a subgroup analysis of the ENGAGE AF-TIMI original study; their aim was to assess the efficacy and safety of edoxaban versus warfarin in patients with atrial fibrillation who had high risk of falling. One of the safety endpoints was fracture; the authors found an increased risk of fractures in the at-risk-of-falling group versus the control group, but there was not a significant difference between bone fractures in the warfarin versus NOAC group. Finally, Norby et al. [56] also compared efficacy and safety of rivaroxaban with dabigatran and warfarin in patients with nonvalvular atrial fibrillation and found a lower risk of hip/pelvic fracture in patients taking rivaroxaban compared to those taking warfarin, but not those taking dabigatran.

Papers comparing fracture risk between VKAs and NOACs as their primary outcome are also scant. Only two observational studies compare fracture risk between both as its primary outcome: one paper by Lau et al. and a short letter to the editor describing a cohort study by Lucenteforte et al. [55]. Lau et al. compared fracture risk in propensity-score-matched individuals with nonvalvular atrial fibrillation taking dabigatran or warfarin [54]. In this study, dabigatran significantly reduced osteoporotic fractures compared to warfarin (0.7 vs. 1.1 per 100 person-years; absolute risk difference per 100 person-years, −0.68 [95% CI, −0.38 to −0.86]). However, an alternative explanation for this study's results has been proposed [59], suggesting that the lower hip plus vertebral fracture risk found in it may be due to not an increased risk with warfarin but to a decreased risk of fracture in the dabigatran group. On the contrary, Lucenteforte et al. [55] carried out a cohort study in which osteoporotic fracture occurrence was assessed in patients taking warfarin, dabigatran, apixaban, or rivaroxaban. Despite the large group of 16,850 patients, the difference in fracture risk between warfarin and the aforementioned NOACs was not significantly different (HR of 1.04 [0.68–1.59] for direct Xa inhibitors; 0.96 [0.56–1.63] for dabigatran). However, the rate of fracture was low overall, and this might have underpowered the study (i.e., only 26/1579.42 fracture/person-years was observed in the NOAC group).

The results of the aforementioned four observational studies were combined in a meta-analysis by Fioderllsi et al [57], finding no significant increase in fracture risk with the use of VKAs versus NOACS. However, in subgroup analysis, the authors did find an increase in fracture risk in two groups: women (pooled OR 1.11, 95% CI 1.02, 1.21) and older VKA users (≥65) (pooled OR 1.07, 95% CI 1.01, 1.14).

In light of these results, it is clear that a RCT comparing fracture risk between NOACs and VKA is needed. Up till then, no solid conclusion can be drawn on the bone-safety profile of NOACs due to the limited and disparate evidence.

## 6. Limitations of the Available Evidence

As expressed earlier, differences in results between studies may be influenced by many confounding factors including the different forms of vitamin K used, baseline dietary vitamin K intake of the included subjects, the level of calcium and vitamin D dietary intake, the use of cosupplements, and differences in baseline characteristics of the population. For instance, in general, Japanese studies use MK-4, European studies use MK-2, and American studies use K1. Also, Japanese studies tend to include more aged population with primary or secondary osteoporosis, with lower vitamin D and calcium levels and thus with a higher baseline fracture risk. This makes extrapolation of results difficult. It is also worth mentioning that most of the cited studies evaluate vitamin K intake or supplements and fracture risk measure of the total number of incident fractures instead of patient fracture. This is key in interpreting the results of these studies; having few patients account for most of the new fractures of a study is not the same as having all of the participants in a study having an incident fracture.

Leaving methodological issues aside, there are other important issues to take into consideration when interpreting studies on vitamin K and bone. For example, ucOC cannot effectively bind mineral to bone. However, studies that manage high levels of carboxylated osteocalcin do not correlate with an increase in BMD. It is possible that effects of vitamin K on BMD are more notable in patients with baseline BMD problems (osteoporosis/osteopenia) or vitamin D-deficient patients (a relation exists between vitamin K and vitamin D). Moreover, the effects of vitamin K on BMD in nondeficient patients are unclear. Whether there is a threshold value or a linear relationship between serum vitamin K values and BMD is unknown. Finally, it is worth mentioning that although most studies assess BMD as the principal endpoint, other bone parameters that reflect bone geometry and resistance should be used to complete bone quality assessment.

## 7. Conclusions

Vitamin K plays an important role in bone health. Low vitamin K intake, low serum vitamin K values, and high levels of ucOC are associated with risk of fracture (especially hip fracture) in observational studies. However, clinical trials do not achieve conclusive results, and thus, there is still controversy over the use of vitamin K1 and K2 supplements. High-quality clinical trials involving patients with low serum vitamin K values and/or low dietary intake are needed to clarify the role of vitamin K in fracture risk.

## Supplementary Materials

Supplementary Materials Main studies on the effect of vitamin K supplements and different bone parameters and fracture. RCT stands for randomized controlled trial.
