# Supplementary material for: Vitamin K and Bone Health: A Review on the Effects of Vitamin K Deficiency and Supplementation and the Effect of Non-Vitamin K Antagonist Oral Anticoagulants on Different Bone Parameters
Source: J Osteoporos. 2019 Dec 31;2019:2069176. doi: 10.1155/2019/2069176 (PMC6955144; doi:10.1155/2019/2069176)
Supplement: Supplementary Materials — Main studies on the effect of vitamin K supplements and different bone parameters and fracture. RCT stands for randomized controlled trial. [file 2069176.f1.docx]

| **Author, year** | **Type of study** | **Population (n)** | **Intervention** | **Results** |
| --- | --- | --- | --- | --- |
| 1. **VITAMIN K SUPPLEMENTS AND BMD.** | | | | |
| Fang et al (2012) | Meta-analysis | 17 studies (3359) | 10 studies K2 (8 MK-4, 2 MK-7)  7 studies K1. | No significant association with BMD at femoral neck. Significant increase of BMD in lumbar spine by 1.27% (CI 95%: 0.47-2.06) only for K2, Asian population, secondary osteoporosis or non-menopausal women. |
| Huan et al (2014) | Meta-analysis | 19 RCT (6759) | Only RCTs, only K2 supplements with a control group. | Significant improvement in middle and long-term vertebral BMD and long-term forearm BMD in postmenopausal women with osteoporosis. |
| Moschonis et al | RCT | Greek postmenopausal women (115) | Control group Vs Three intervention group: calcium+vitamin D3, calcium+ vitamin D3+K1, calcium+vitamin D3+K2. | Significant increase in total BMD in all three groups compared with controls.  Additional benefit for lumbar BMD in the groups who took either K1 or K2. |
| 1. **VITAMIN K SUPPLEMENTS AND OTHER BONE PARAMETERS.** | | | | |
| Knapen et al (2007) | RCT | Dutch non-osteoporotic postmenopausal women without hormonal replacement therapy (325). | 45 mg/day MK-4 or placebo for three years. | MK-4 supplementation did not significantly increase BMD at the hip, but bone mineral content and femoral neck width were both significantly increased. |
| Knapen (2013) | RCT | Healthy postmenopausal women (244). | 180 μg MK-7/day for three years. | Significant decrease in BMD decline at lumbar spine and femoral neck, but not at total hip.  Increased bone strength (measured as compression, bending and impact strength). |
| 1. **VITAMIN K SUPPLEMENTS AND FRACTURE.** | | | | |
| Cockayne (2006) | Meta-analysis | 13 RCT. | K1 (2 studies) or Mk-4 (11 studies) supplements. Effect on BMD and fractures.  - Only 7 RCT reporting fracture, all Japanese, and all using K2. | - All thirteen trials except one showed a decrease in bone loss (measured by BMD).  - MK-4 supplements caused a significant reduction in all fracture types. |
| Inoue (2009) | RCT | Japanese osteoporotic postmenopausal women (4378). | Three-year calcium monotherapy or bitherapy with MK-4. | No significant differences in fractures, only post-hoc analysis showed decrease in vertebral fractures in women who had 5 or more prevalent fractures. |
| Cheung (2008) | RCT | Postmenopausal Canadian women with osteopenia (440). | K1 5mg/day versus placebo. | No changes in BMD at lumbar spine or total hip (primary outcome).  Decrease in clinical vertebral fractures (9 versus 20, p 0.04). |

*Table 1.* Main studies of the effect of Vitamin K supplements on different bone parameters and fracture risk. RCT stands for randomized controlled trial, BMD for bone mineral density, MK for menaquinone.
